# Carnosine Potentiates Doxorubicin-Induced Cytotoxicity in Resistant NCI/ADR-RES Cells by Inhibiting P-Glycoprotein—In Silico and In Vitro Evidence

**DOI:** 10.3390/molecules27217383

**Published:** 2022-10-30

**Authors:** Mohamed A. Morsy, Mahmoud Kandeel, Ahmed R. N. Ibrahim, Seham A. Abdel-Gaber, Shery Jacob, Katharigatta N. Venugopala, Pottathil Shinu, Mahmoud El-Daly

**Affiliations:** 1 Department of Pharmaceutical Sciences, College of Clinical Pharmacy, King Faisal University, Al-Ahsa 31982, Saudi Arabia; 2 Department of Pharmacology, Faculty of Medicine, Minia University, El-Minia 61511, Egypt; 3 Department of Biomedical Sciences, College of Veterinary Medicine, King Faisal University, Al-Ahsa 31982, Saudi Arabia; 4 Department of Pharmacology, Faculty of Veterinary Medicine, Kafrelsheikh University, Kafr El-Sheikh 33516, Egypt; 5 Clinical Pharmacy Department, College of Pharmacy, King Khalid University, Abha 61441, Saudi Arabia; 6 Department of Biochemistry, Faculty of Pharmacy, Minia University, El-Minia 61511, Egypt; 7 Department of Pharmaceutical Sciences, College of Pharmacy, Gulf Medical University, Ajman 4184, United Arab Emirates; 8 Department of Biotechnology and Food Science, Faculty of Applied Sciences, Durban University of Technology, Durban 4000, South Africa; 9 Department of Biomedical Sciences, College of Clinical Pharmacy, King Faisal University, Al-Ahsa 31982, Saudi Arabia; 10 Department of Pharmacology & Toxicology, Faculty of Pharmacy, Minia University, El-Minia 61511, Egypt

**Keywords:** P-glycoprotein, carnosine, multidrug resistance, doxorubicin, molecular dynamics simulations, cancer chemotherapy

## Abstract

The activity of the P-glycoprotein (P-gp) transporter encoded by the ABCB1 gene confers resistance to anticancer drugs and contributes to cancer-related mortality and morbidity. Recent studies revealed the cytotoxic effects of the endogenous dipeptide carnosine. The current study aimed to investigate the role of carnosine as a potential inhibitor of P-gp activity. We used molecular docking and molecular dynamic simulations to study the possible binding and stability of carnosine-P-gp interactions compared with verapamil. In vitro assays using doxorubicin-resistant NCI/ADR-RES cells were established to test the effects of carnosine (10–300 µM) on P-gp activity by the rhodamine-123 efflux assay and its effect on cell viability and doxorubicin-induced cytotoxicity. Verapamil (10 µM) was used as a positive control. The results showed that carnosine binding depends mainly on hydrogen bonding with GLU875, GLN946, and ALA871, with a higher average Hbond than verapamil. Carnosine showed significant but weaker than verapamil-induced rhodamine-123 accumulation. Carnosine and verapamil similarly inhibited cell viability. However, verapamil showed a more significant potentiating effect on doxorubicin-induced cytotoxicity than a weaker effect of carnosine at 300 µM. These results suggest that carnosine inhibits P-gp activity and potentiates doxorubicin-induced cytotoxicity at higher concentrations. Carnosine might be a helpful lead compound in the fight against multidrug-resistant cancers.

## 1. Introduction

According to the WHO records, cancer ranks second as a cause of death worldwide, with a death toll of 10 million in 2018 alone [1]. The global estimates of cancer are devastating, with nearly 18 million new cases in 2018 and an alarming incidence of 37 million new cases by 2040, with the highest rates in low- and medium-income countries [1]. Besides the lack of adequate therapies and late discovery in many cases, the development of multidrug resistance (MDR) contributes to this high mortality. One important mechanism by which cancer cells resist cytotoxic drugs is increasing the expression of specific efflux pumps that shuttle organic molecules to the outside of the cell across the plasma membrane, preventing their intracellular accumulation to cytotoxic levels and ultimately leading to therapeutic failure [2,3]. These transmembrane efflux pumps include P-glycoprotein (P-gp), a member of the ATP-binding cassette (ABC) family of transporters that are associated with cancer drug resistance [4,5]. Thus, targeting these transporters would increase treatment efficacy and decrease cancer-associated mortality [3].

The activity of the ABC family of transporters protects normal body cells from chemical insults by shuttling xenobiotic molecules across the cell membrane [6]. Contrarily, the expression and function of such transport systems largely determine the susceptibility of cancer cells to chemotherapeutic agents [2,3]. The increased expression and/or activity of P-gp, encoded in humans by the ABCB1 gene, confers resistance to anticancer drugs [4,5]. Clinically, tumors overexpressing P-gp are more resistant to chemotherapy and correlate with poor patient prognosis [7,8]. Nutritional factors, health state, and the signaling milieu in the tumor microenvironment are among the factors that determine the expression of MDR proteins, including P-gp [9,10,11], which might modify the expected treatment outcomes. Many anticancer drugs, including doxorubicin, are substrates of P-gp [12]. Thus, inhibiting such transporter proteins is gaining interest as a potential therapeutic approach to overcome resistance to anticancer chemotherapy [3,6,12,13,14,15,16].

With the current understanding of the domain structure of P-gp [15,17], in silico modeling studies provide an essential tool to discover potential P-gp-active molecules that can improve anticancer therapeutics. We have previously illustrated the plausibility of molecular docking and molecular dynamics (MD) simulation studies in predicting P-gp inhibitory functions [6,13]. These in silico approaches were confirmed by studying cancer cell lines in vitro [6,13]. The current work investigates the role of carnosine, an endogenous dipeptide, as a potential inhibitor of P-gp activity. We adopted two approaches to achieve this aim: an in silico study involving MD simulations and molecular docking and an in vitro one testing the inhibition of cellular P-gp by carnosine and its effect on doxorubicin-induced cytotoxicity.

## 2. Results

### 2.1. MD Simulation and Docking Studies

#### 2.1.1. Molecular Docking

Docking studies were performed to assess the binding properties of carnosine with P-gp. The standard Glide docking protocol (SP) was used. Carnosine showed a docking score of −5.54, compared with −7.38 and −9.1 for verapamil and the co-crystalized ligand (paclitaxel), respectively. These values reflect the interactions of each compound with P-pg, which is affected by the size and volume of interactions of each compound with the protein. In terms of ligand efficiency, measured as the binding energy normalized to the number of atoms in the ligand, carnosine showed the most favorable binding profile, evidenced by the lowest ligand efficiency values (Table 1). In addition, the binding of carnosine was supported by Hbond and the highest Coulombic forces scores.

#### 2.1.2. The Root Mean Square Deviation (RMSD)

The average change in displacement of a group of atoms with respect to a reference frame is calculated in terms of the RMSD. During the simulation, monitoring the protein’s RMSD can provide insight into its structural aspects as a unit. Changes within 1–3 Å are reasonably acceptable for globular proteins. On the other hand, higher values imply that the protein changes significantly in shape during the simulation.

**Figure 1:**
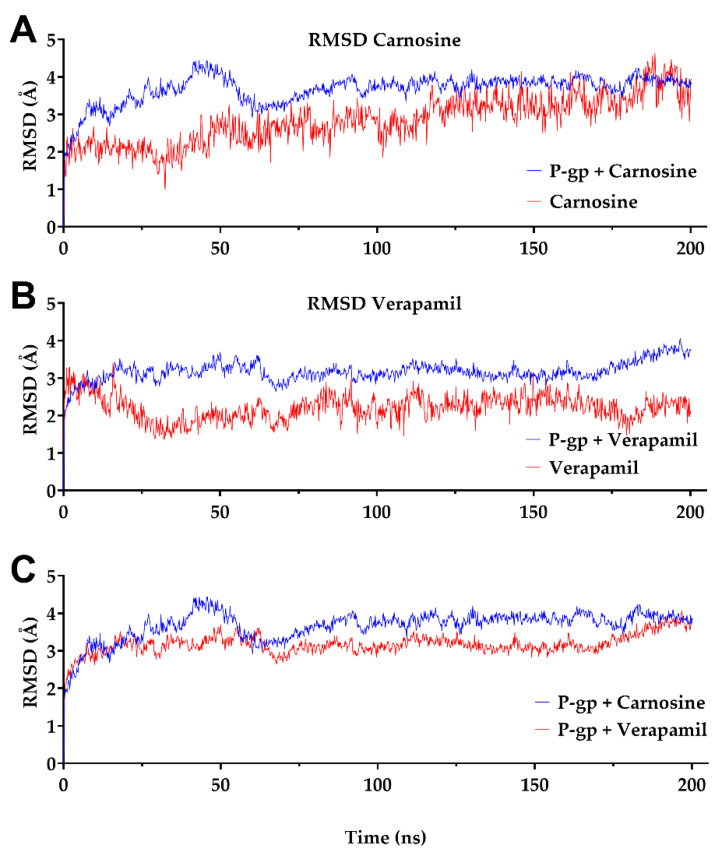
The root mean square deviation (RMSD) of the alpha carbon atoms of P-glycoprotein (P-gp) bound with carnosine or verapamil during 200 ns molecular dynamics simulation. In (**A**) and (**B**), the protein bound with ligands RMSD is shown in blue, while the RMSD values of the ligand [(**A**) carnosine; (**B**) verapamil] are shown in red. (**C**) Compares the RMSD values of the alpha carbon atoms of P-gp bound with carnosine (blue) or verapamil (red) during 200 ns simulation. The PDB file 6QEX was selected as a template of P-gp and used for molecular dynamics simulations in the absence or presence of ligands (carnosine or verapamil) throughout 200 ns using the Desmond package of Schrödinger, as described in further detail in Section 4.

The protein and ligand RMSD values reveal stable binding complexes of carnosine and verapamil, which is used here as a standard P-gp inhibitor (Figure 1). All structures equilibrated within 70 ns, and the RMSD values stabilized until the end of the simulation. The ligand RMSD values for either carnosine or verapamil (red color) were lower than those of the protein RMSD (Figure 1A,B; blue color), indicating stable binding of carnosine and verapamil with the binding site during the simulation time. As shown in Figure 1C, RMSD values of the P-gp-verapamil complex were lower than that of the P-gp-carnosine complex, indicating a more stable interaction between P-gp and verapamil than with P-gp and carnosine.

#### 2.1.3. The Root Mean Square Fluctuation (RMSF)

**Figure 2:**
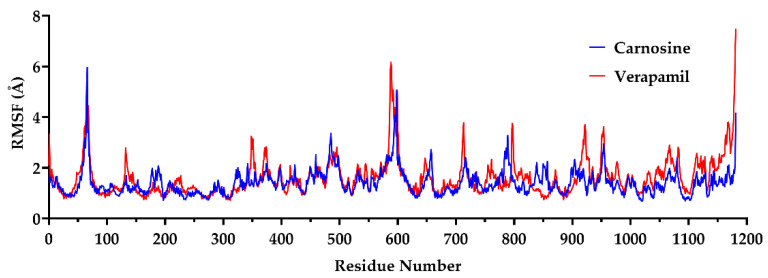
The root mean square fluctuation (RMSF) of carnosine-(blue line) and verapamil-bound (red line) P-glycoprotein (P-gp) during 200 ns molecular dynamics simulation. The PDB file 6QEX was selected as a template of P-gp and used for molecular dynamics simulations in the presence of either carnosine or verapamil for 200 ns using the Desmond package of Schrödinger, as described in further detail in Section 4.

Unlike the RMSD, which indicates structural changes in the whole protein, the RMSF calculates the movement of each residue in the protein. Analysis of the protein RMSF values revealed almost similar residue fluctuations in the presence of either carnosine or verapamil (Figure 2). The residues in the range of 560–620 showed the highest fluctuation values.

#### 2.1.4. The Number of Hydrogen Bonds (Hbonds)

**Figure 3:**
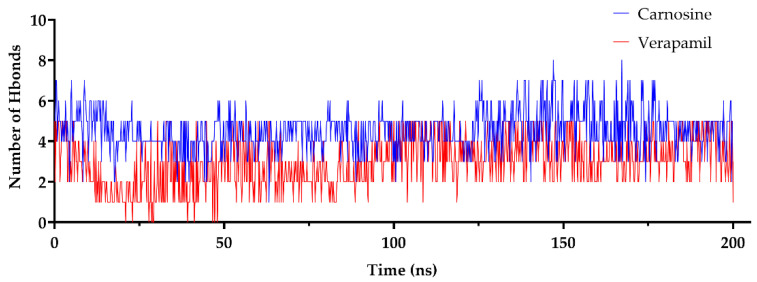
The number of hydrogen bonds (Hbonds) formed between P-glycoprotein (P-gp) and carnosine or verapamil during a 200 ns molecular dynamics simulation. The PDB file 6QEX was selected as a template of P-gp and used for molecular dynamics simulations in the presence of either carnosine or verapamil for 200 ns using the Desmond package of Schrödinger, as described in further detail in Section 4.

The number of Hbonds formed between P-gp and either carnosine or verapamil was measured during the MD simulation, as shown in Figure 3 and Table 2. Carnosine interactions with P-gp resulted in a higher maximum number of Hbonds (8) compared to verapamil (5). Moreover, the average number of Hbonds during the 200 ns simulation time was 4.45 and 2.98 for carnosine and verapamil, respectively. These findings show the higher contribution of Hbond formation in carnosine binding to P-gp as opposed to the binding pattern of verapamil.

#### 2.1.5. The Length of Potential Bonding Interactions

**Figure 4:**
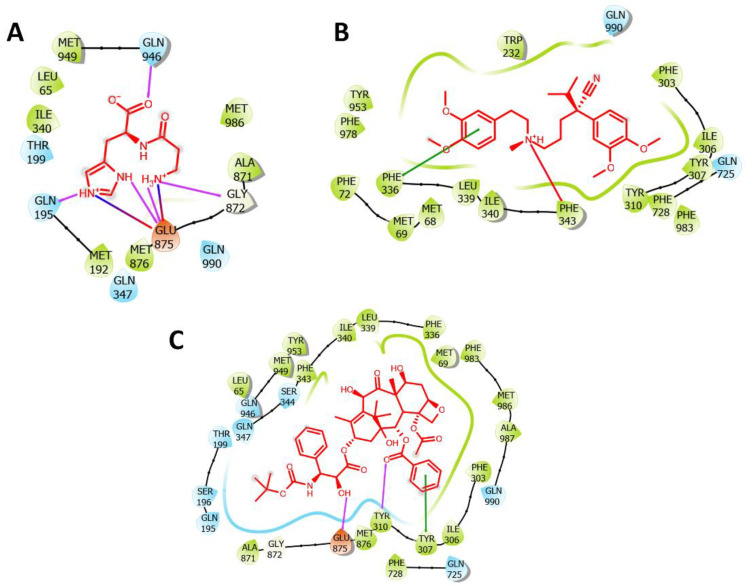
The ligand interactions of carnosine, verapamil, and co-crystalized ligand (paclitaxel) with P-glycoprotein (P-gp). (**A**) Carnosine docking site with P-gp. (**B**) Verapamil docking site with P-gp. (**C**) Paclitaxel docking site with P-gp. Hydrogen bonds are shown in purple, stacking interaction in green, and salt bridges in multicolored sticks.

**Figure 5:**
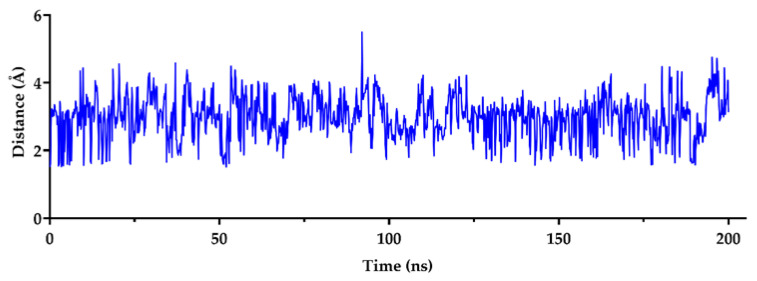
The distance (Å) of the bonding interactions of carnosine with P-glycoprotein GLU875.

The data in Figure 4 show the interactions of carnosine, verapamil, and the co-crystalized ligand with the amino acid residues in its docking site on the P-gp molecule. Carnosine forms four Hbonds with the side chains of GLU875 and GLN946 and the backbone of GLY872 with a stacking interaction with GLU875. In addition, salt bridges were also evident with GLU875. Carnosine interactions were significantly more diversified than those observed with verapamil and paclitaxel, which displayed less hydrogen bonding and a lack of salt bridges. Based on the described interactions with P-gp binding site, we further investigated the stability of the carnosine binding complex with P-gp by tracing the Hbond distance between the imidazole ring of carnosine and the side chain of GLU875 during the 200 ns simulation. The results in Figure 5 and Table 3 suggest a stable binding conformation between carnosine and GLU875, as evidenced by the stable and fixed length of Hbond. The summary statistics of carnosine binding with the side chain of GLU875 (Table 3) show an average length of 2.9 Å and low SD, indicating stable binding.

### 2.2. Effect of Carnosine on Rhodamine-123 Efflux

**Figure 6:**
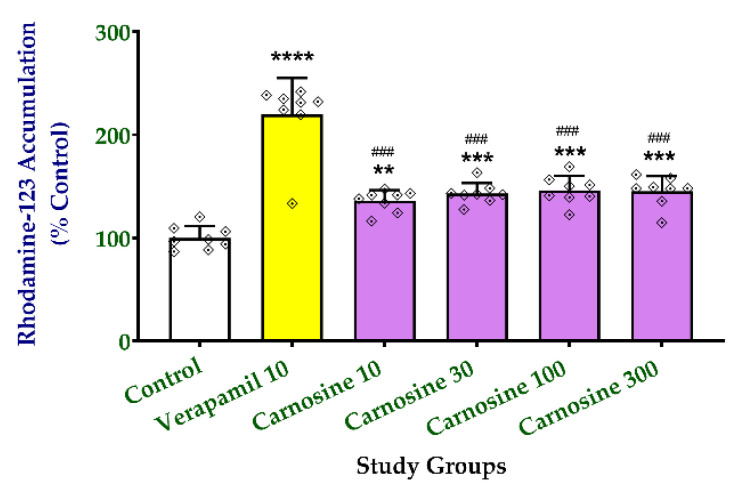
Carnosine inhibits P-glycoprotein activity in vitro and decreases the efflux of rhodamine-123. The rhodamine-123 efflux assay was carried out on NCI/ADR-RES cells as described in Methods. Cells were incubated with rhodamine-123 (1 μM) for one hour in the absence (white bar) or presence of 10 μM verapamil (yellow bar) or increasing concentrations of carnosine (10–300 μM, purple bars). Data (mean ± SD, n = 8) represent the % rhodamine-123 accumulated inside the cells relative to untreated controls. Data were analyzed with one-way ANOVA followed by the Tukey test for comparison between groups. **, ***, **** indicate significant differences from control cells at *p* < 0.01, 0.001, and 0.0001, respectively; ### indicates significant differences from the verapamil-treated cells at *p* < 0.001.

To test the ability of carnosine to inhibit P-gp activity, we carried out the rhodamine-123 efflux assay comparing the effects of increasing carnosine concentrations (10–300 μM) to the effect of 10 μM verapamil, a reference standard inhibitor of P-gp. Verapamil treatment of NCI/ADR-RES cells induced the highest intracellular rhodamine-123 accumulation (219.5 ± 35.59%) compared with the control untreated cells (Figure 6). Compared with controls, treatment of the NCI/ADR-RES cells with carnosine also significantly increased the accumulation of the fluorescent dye to 135.8–145.1% at the tested concentrations (10–300 μM). However, all the carnosine-treated cells accumulated significantly lower concentrations of rhodamine-123 than the verapamil-treated cells. There were no significant differences between the data obtained at different concentrations of carnosine. These data indicate that carnosine inhibited the P-gp activity to a lesser extent than the standard drug verapamil (10 μM).

### 2.3. Effect of Carnosine on Cell Viability and Doxorubicin-Induced Cytotoxicity

**Figure 7:**
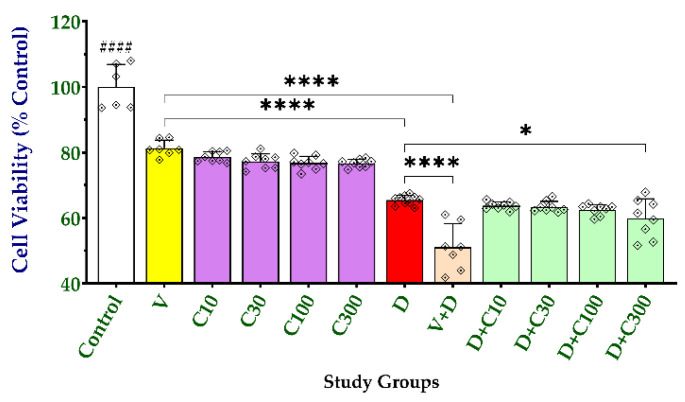
Effect of carnosine on cell viability and doxorubicin-induced cytotoxicity. NCI/ADR-RES cells were initially seeded at 1.0 × 10^4^ cells/well in DMEM and allowed to grow for 24 h before starting different treatments for another 24 h without or with carnosine (10–300 μM), verapamil (10 μM), doxorubicin (1 μM), or combinations of doxorubicin (1 μM) with either carnosine (10–300 μM) or verapamil (10 μM). The MTT assay was carried out as described in Methods. Data (mean ± SD of at least six replicates) represent the percentage of viable cells after each treatment relative to the untreated controls. Data were analyzed with one-way ANOVA followed by the Tukey test for comparison between groups. *, **** indicate significant differences between the selected treatments at *p* < 0.05 and 0.0001, respectively; #### indicates significant differences between the control group and all other treatment groups at *p* < 0.0001. Verapamil (V); carnosine (C); doxorubicin (D).

We evaluated the effect of increasing carnosine concentrations (10–300 μM) on cell viability and doxorubicin-induced toxicity compared with verapamil (10 μM). Interestingly, all carnosine concentrations, like verapamil, significantly reduced cell viability to the same extent (18.7% for verapamil; 21.4–23.3% for carnosine concentrations) compared with the untreated cells (Figure 7). Figure 7 shows no significant differences in cell viability between verapamil- or carnosine-treated NCI/ADR-RES cells or between the different carnosine concentrations.

Treating the doxorubicin-resistant NCI/ADR-RES cells with doxorubicin (1 μM) for 24 h significantly reduced the viability by 34.6 ± 1.46%. This cytotoxic effect was further increased by concomitant treatment with verapamil (48.9 ± 7.18%, *p* < 0.0001). Carnosine at 10–100 μM did not significantly change doxorubicin-induced cytotoxicity. On the other hand, the highest tested concentration of carnosine (300 μM) significantly, but only slightly, potentiated doxorubicin-induced reduction in cell viability (40.1 ± 5.92, *p* < 0.05). There were no significant differences between the data obtained at different concentrations of carnosine. These data indicate that carnosine-mediated inhibition of cell viability is comparable to that induced by verapamil and that it potentiates doxorubicin cytotoxicity only at higher concentrations (300 μM).

## 3. Discussion

Cancer ranks second to heart disease as a cause of death worldwide. Resistance of the cancer cell to chemotherapy exaggerates the problem of increased cancer-associated mortality and morbidity [1]. The increased expression and activity of P-gp diminishes drug delivery to cellular targets and increases the MDR of cancers [2,4,5]. Indeed, clinical evidence implicates P-gp with therapy failure and poor prognosis [7,8]. Thus, finding new P-gp inhibitors would augment the current anticancer therapeutic approaches [3,12,14,15]. On the other hand, recent studies showed the anticancer activity of carnosine [18,19]. Thus, the present work investigated the potential inhibitory effect of carnosine on P-gp activity using in silico and in vitro approaches.

The results of docking studies and MD simulations revealed the ability of carnosine to inhibit P-gp, which was confirmed by the results of in vitro studies. The low RMSD and stable Hbonds observed with carnosine in the modeling studies suggest a steady binding profile with P-gp. On average, the interactions between carnosine and P-gp involved the formation of 4.45 Hbonds. Moreover, tracking of carnosine binding through Hbonds during a 200 ns simulation revealed stable and distant interactions with GLU875 of P-gp. Hydrogen bonding and stacking interactions were the most observed forces contributing to carnosine binding to P-gp.

On the other hand, the lower RMSD values found with verapamil, as compared with carnosine, suggest stronger verapamil-P-gp binding. However, unlike carnosine, verapamil showed lower hydrogen bonding and stacking interactions. These results suggest that different binding mechanisms stabilize the carnosine- or verapamil-mediated interactions with P-gp. The docking studies showed the presence of salt bridges with carnosine, which supports the potential strong binding of carnosine with P-gp. In addition, the binding site of verapamil in P-gp comprised PHE303, TYR310, PHE343, and LEU339, which supports previous findings showing the importance of these residues in P-gp inhibition [20,21]. These results are supported by previous observations showing P-gp as a low-specificity transporter capable of handling a wide range of substrates [6,17,22].

In the present study, carnosine increased the accumulation of rhodamine-123 in NCI/ADR-RES cells, a doxorubicin-resistant breast cancer cell line known for its high expression of P-gp [23]. This rhodamine efflux assay is a standard tool for studying P-gp activity [6,15,24]. The well-known inhibitor of P-gp activity, verapamil, dramatically inhibited rhodamine-123 efflux in the current study, which aligns with previous findings [6,13]. Together with the data observed in silico by MD simulations and docking studies, these results suggest stable interactions between P-gp and carnosine or verapamil.

Given its P-gp inhibitory effects, we asked whether carnosine affects cell viability and whether it could potentiate the cytotoxic effects of doxorubicin. Culturing the NCI/ADR-RES cells with different carnosine concentrations resulted in significant cell mortality, which is supported by recent reports of carnosine-induced cytotoxicity in cancer models [18,19]. In one study [19], the anticancer effects of carnosine were mediated by increased autophagy and necroptosis signaling and reduced angiogenesis. In addition, the results of recent research showing that carnosine and an analog containing the L, rather than the D, amino acids both inhibit patient-derived glioblastoma cells but not fibroblasts [18] are intriguing. The authors concluded that the imidazole moiety was essential to the carnosine-mediated cytotoxicity. However, the carnosine concentrations used in these studies were 10–15 mM [19] and 50 mM [18] compared with 10–300 µM in the current work. Thus, one should be cautious when explaining these results. Nonetheless, in support of such observations, the docking and MD simulations in the present study suggested strong interactions between the imidazole ring of carnosine and GLU875. Together, these results support the cytotoxic activity of carnosine we observed here.

However, the current results do not explain the lack of a concentration-dependent inhibition response of carnosine on cell viability since all the used concentrations induced comparable reductions. A similar pattern was also observed in the rhodamine-123 efflux assay. These observations might suggest a time- rather than a concentration-dependent effect of carnosine or that a maximal or near-maximal effect was achievable at the lower concentration (10 µM) tested in the current work. Another possibility could be that much higher concentrations were required to show a concentration-effect relationship, especially since the anticancer effects observed by others were obtained with carnosine in the millimolar range [18,19], unlike the micromolar range we used in the current study. Further research is thus required to unravel this promiscuity.

The current results revealed only a weak possible potentiation by carnosine of doxorubicin-induced cytotoxicity at 300 µM. The lower concentrations of carnosine (≤100 µM) did not increase the toxicity of doxorubicin. As expected, all cells receiving combined carnosine and doxorubicin treatments were less viable than the corresponding carnosine-only treatments. Although verapamil alone decreased cell viability to levels comparable to carnosine treatments, the combination of verapamil with doxorubicin showed the highest cytotoxic effects. The higher doxorubicin-potentiating effects of verapamil compared with carnosine are in line with our results showing the more potent effect of verapamil in the P-gp inhibition assay and supported by previous findings [6,13].

The efflux of xenobiotics is a powerful mechanism leading to MDR during cancer chemotherapy [2,3]. Unfortunately, like many anticancer agents, doxorubicin suffers extrusion through the cell membrane by the action of P-gp [12,25,26], dramatically diminishing its efficacy. Scientific efforts to overcome such problems include chemical modifications or novel delivery systems to make drugs less vulnerable to P-gp or empower them with inhibitory effects [16,27,28,29,30]. Equally important, the continued search for natural P-gp inhibitors showed promising results [6,12,13].

Inhibiting the ABC transporters might compromise cellular protection against toxic chemicals; however, it constitutes a great opportunity in the fight against MDR cancer [5,7,26]. Accumulating evidence implicates P-gp in clinical cancer resistance and increased mortality [4,5,7,8]. The fact that multiple factors affect the activity and expression of these transporters adds further importance to the research in this field. Modulators of P-gp activity and expression include nutritional factors such as salt intake [9,11], patient factors and genetic makeup [2,31], and the activation of various signaling pathways in cancer compared with normal cells [5,10,32]. Thus, with the help of current knowledge on the detailed domain structure of P-gp [15,17] and advanced in silico screening technologies [6,29], the search for better P-gp inhibitors shall continue to increase anticancer therapeutic efficacy and decrease patient suffering.

## 4. Materials and Methods

### 4.1. Protein Preparation, Docking, and MD Simulation

The ligand preparation, docking, and preparation for MD simulation were carried out as previously described, with slight modifications [15]. We selected the PDB file 6QEX [17] as a template for computational studies [15]. The structure comprised P-gp in combination with paclitaxel, a potent anticancer drug. The reference inhibitor verapamil was selected and prepared as previously done [15]. Paclitaxel was removed from its site, which was used to dock carnosine and verapamil using the software and the protocol implemented in our previous P-gp docking work [13].

Desmond, a package from Schrödinger (New York, NY, USA), was used to run MD simulations for 200 ns. The System Builder tool was used to prepare all the systems. Before system creation, the protein was introduced to a DPPC (325 k) membrane. TIP3P was chosen as the Solvent Model having an orthorhombic box (Transferable Intermolecular Interaction Potential 3 Points). In the simulation, the OPLS 2005 force field was employed. Counter ions were added to the models to make them neutral. NaCl 0.15 M was added to replicate physiological circumstances. For the whole simulation, the NPT ensemble (Isothermal-Isobaric: moles (N), pressure (P), and temperature (T) are preserved) with 300 K temperature and 1 atm pressure was used. Before the simulation, the models were loosened. The trajectories were stored every 100 ps for examination, and the simulation stability was determined by measuring the peptide RMSD over time. The trajectories were analyzed for RMSF, the radius of gyration (RoG), and protein-ligand Hbonds and distances.

### 4.2. Rhodamine-123 Efflux Assay

Assessment of P-gp function was carried out in doxorubicin-resistant NCI/ADR-RES cells according to a previously described protocol [33]. Cells were treated with rhodamine-123 (1 μM) at 37 °C for one hour in the presence or absence of carnosine at increasing concentrations (10–300 μM). Verapamil (10 μM) was used as a standard P-gp inhibitor [6]. After the one-hour incubation, cells were carefully washed with phosphate-buffered saline and lysed. The concentration of rhodamine-123 in the lysate, which reflects its intracellular content, was measured using a spectrofluorometer (λ_excitation_ = 485 nm; λ_emission_ = 535 nm). The mean percentage of rhodamine-123 accumulation was calculated relative to the control untreated cells. The fluorescence emitted by a sample was divided by that of the control, and the product was multiplied by 100.

### 4.3. Cell Viability and Cytotoxicity Assay

We assessed the cell viability of doxorubicin-resistant NCI/ADR-RES cells in 96-well plates using the 4,5-dimethyl-2-[3-(3-phenyl-2H-tetrazol-3-ium-5-yl)phenyl]-1,3-thiazole bromide (MTT) assay as previously described [6,13]. Briefly, cells (1.0 × 10^4^ cells/well) were cultured for 24 h in DMEM in 96-well plates. Wells were treated with or without carnosine (10–300 μM), verapamil (10 μM), doxorubicin (1 μM), or combinations of doxorubicin (1 μM) with either carnosine (10–300 μM) or verapamil (10 μM). All treatments were maintained for another 24 h, after which 15 μL of MTT (5 mg/mL in phosphate-buffered saline) was added to each well. After incubating the cells with MTT for 4 h at 37 °C in the dark, DMSO (100 μL) was added to each well for 10 min to dissolve the formed formazan. A microplate reader was used to measure the absorbance at 540 nm.

### 4.4. Data Analysis

All cell culture data were expressed as the mean percent change from controls ± SD. Data were statistically analyzed using one-way analysis of variance (ANOVA) followed by the Tukey post hoc test for multiple comparisons. GraphPad Prism software version 8.00 (San Diego, CA, USA) was used for the different analyses. Differences between means were significant at *p* < 0.05.

## 5. Conclusions

P-gp inhibition is a potential target to enhance anticancer drug delivery and efficacy. We have shown by in silico and in vitro approaches that carnosine can inhibit P-gp activity, decrease cell viability, and enhance doxorubicin-induced toxicity at higher concentrations. The docking and MD simulation studies showed that Hbond formation between carnosine and the amino acids GLU875, GLN946, and ALA871 and a stacking interaction with GLU875 are essential to the stability of the carnosine-P-gp complex.

## Figures and Tables

**Table 1:** The docking score and binding properties of carnosine, verapamil, and the co-crystalized ligand.

	Docking Score	Ligand Efficiency	Hbond	Lipo Score	Ecoul
Carnosine	−5.54	−0.32	−0.547	−0.079	−22.234
Verapamil	−7.38	−0.22	−0.64	−3.86	−6.18
Co-crystalized ligand	−9.1	−0.157	−0.219	−4.169	−6.169

**Table 2:** Summary of hydrogen bond statistics of the interaction between carnosine or verapamil with P-glycoprotein.

	Minimum	Maximum	Mean	SD
Carnosine	1	8	4.45	1.01
Verapamil	0	5	2.98	1.23

**Table 3:** Summary of distance statistics of the binding of carnosine with P-glycoprotein GLU875.

	Minimum	Maximum	Mean	SD
Carnosine	1.51	5.49	2.9	0.55

## Data Availability

Data are contained within the article or available upon reasonable request from the corresponding author.
